# Survey data on determinants and barriers to the adoption of improved maize seeds among smallholder farmers in the Katangese Copperbelt

**DOI:** 10.1016/j.dib.2025.111689

**Published:** 2025-05-19

**Authors:** Arsene Mushagalusa Balasha, Angelo Fiore, Mazinga Kwey Michel, Alex Nyumbaiza Tambwe

**Affiliations:** a Agricultural Economics, University of Lubumbashi, PO Box 1825, Lubumbashi, Democratic Republic of the Congo; b Department of Sociology and Anthropology, University of Lubumbashi, PO Box 1825, Lubumbashi, Democratic Republic of the Congo

**Keywords:** Agricultural innovations, Maize improved varieties, Haut-Katanga, Household survey, Farmers’ profile

## Abstract

This dataset describes survey data on the drivers and constraints of adopting improved maize varieties among smallholder farmers in the mining region of Haut-Katanga, Democratic Republic of the Congo. The household survey was conducted using a structured questionnaire administered to 300 smallholder farmers randomly selected from villages around Lubumbashi and Kipushi, including the agricultural zone of Sambwa.

Information collected from maize farmers includes: (1) demographic profile (gender, age, education, household size); (2) socioeconomic and farm characteristics (land tenure, cropland size, farming experience, financial support, off-farm employment, obstacles to using improved seeds, reasons for using improved seeds, and concerns about climate change); (3) farmers' networks (membership in agricultural associations, Village Savings and Loan Associations, sources of information, and seed supply channels); and (4) farmers' willingness to recommend these seeds to peers and barriers they face in accessing them.

The raw data and codebook are available on Mendeley Data. The data will be reused in a detailed article to provide a deeper understanding of the factors and challenges associated with the uptake of agricultural innovations among smallholder farmers in the Katangese Copperbelt.

Specifications Table

**Table:** 

Subject	Agricultural economics, Agronomy, and crop Science
Specific subject area	Quantitative data socio-economic characteristics of farmers, Sources of information adoption of agricultural technologies agricultural production food security Haut Katanga
Type of data	Numerical and categorical data are presented in tables, Figures, and Excel file
Data collection	Data were acquired through face-to-face interviews with 300 randomly selected smallholder farmers, using a structured questionnaire. Interviews were conducted in Kiswahili. Data collected were recorded in an Excel sheet.
Data source location	A household survey was conducted in the mining region of Haut-Katanga in the territory of Kipushi, around the city of Lubumbashi southeastern DR Congo. Kipushi extends between 11° 46′ 54″ South, Latitude, 27° 14′ 42″ East Longitude, Altitude **:** 1 329 m
Data accessibility	Repository name: **Mendeley Data** Data identification number: doi: 10.17632/by7b2dj2fh.1 Direct URL to data: https://data.mendeley.com/datasets/by7b2dj2fh/1
Related research article	*None*

## 1 Value of the Data

• The dataset is crucial for understanding the drivers and barriers to adopting agricultural technologies among smallholder farmers in the Katangese Copperbelt. It provides valuable information to diverse stakeholders, including policymakers, extension officers, non-governmental organizations, agronomists, and plant breeders, to develop programs and inform policies to support smallholder farmers and enhance agricultural production in the Katanga region.
• The dataset also offers valuable insights that can guide decisions and strategies for strengthening the resilience of maize smallholder farmers to environmental challenges in the Katangese Copperbelt, a region where climate change and mining pollution pose significant threats to agricultural production.
• The dataset enables a gender-based assessment of the use of improved seeds compared to local varieties, shedding light on the sources of information and supply channels available to smallholder farmers. Such information is crucial for supporting the maize sector in the Katanga region.
• In the study region, where maize is both a staple food and a key income-generating crop for a large portion of the population, the dataset is of significant relevance. It can be used for a variety of purposes, including local and regional planning, community development initiatives, livelihood promotion programs, and research at both local and regional levels.
• Researchers studying the adoption of agricultural innovations in different regions can leverage this dataset for comparative analysis to identify both common and region-specific determinants of improved seed adoption. Furthermore, the dataset bridges the fields of agricultural economics, rural development, climate change adaptation, and policy analysis, making it an essential resource for interdisciplinary research.

## 2 Background

In the Katangese Copperbelt, maize is one of the most widely cultivated crops among smallholder farmers, serving as both a staple food source and an income-generating crop. However, yields per hectare remain significantly lower than those achieved by farmers in neighboring regions, such as Zambia [1,2]. Kimuni and colleagues report that smallholder farmers in Haut-Katanga province produce barely one ton per hectare, while large-scale farms achieve up to six tons [2,3].

In recent years, the Faculty of Agriculture at the University of Lubumbashi, the National Institute for Agricultural Study and Research of the Democratic Republic of Congo, and seed companies including PANNAR Seed and SEED CO Group have developed and distributed improved maize varieties adapted to Katanga's edaphoclimatic conditions [2,3]. Nevertheless, the key drivers of and barriers to adoption by smallholder farmers remain poorly understood.

The primary motivation behind compiling this dataset is to examine the factors influencing farmers’ adoption of improved seeds to identify those that can serve as levers for promoting agricultural innovation in the region. This dataset serves as an empirical analysis tool that can inform decision-making in agriculture, development planning, and initiatives to strengthen the resilience of smallholder farming to climate change. This study uses survey data from smallholder farmers to identify the socioeconomic and institutional factors affecting the adoption of improved maize seeds in the Katangese Copperbelt. These findings provide actionable insights for researchers, seed suppliers, and policymakers to enhance support for smallholder farmers.

## 3 Data Description

The dataset presented in this study originates from a survey conducted among smallholder farmers cultivating maize in the territory of Kipushi, including the agricultural zone of Sambwa in the province of Haut-Katanga, southeastern Democratic Republic of Congo. We collected survey data on the drivers, motivations, and barriers influencing smallholder farmers’ adoption of improved maize seeds. The respondents’ sociodemographic characteristics included gender, age, education, maize farming experience, and household size. Socioeconomic data covered membership in farmers' groups and village savings and loan associations, land tenure, cropland size, off-farm employment, other crops cultivated, recent agricultural training, seed types used in the past three years, reasons for—and barriers to—adoption, and sources of seed-related information.

To predict the adoption of improved maize seeds, the binary logistic regression model uses a dependent variable based on farmers’ responses to whether they have consistently used improved maize seeds in their fields over the past three years (“yes” or “no”). Socioeconomic and demographic variables serve as predictors, as demonstrated in numerous studies [[4], [5], [6]].

The survey questionnaire, the dataset, and the codebook describing the variables are publicly available in the Mendeley Data repository: https://data.mendeley.com/datasets/by7b2dj2fh/1.

### 3.1 Socioeconomic and demographic characteristics of farmers in the Katangese Copperbelt

The sociodemographic characteristics of the participants are illustrated in Table 1.

**Table 1:** Sociodemographic data of maize farmers in Katanga Copperbelt.

Characteristics	Category	Frequency	Percentage	Gender differences	
				χ2	*p*-value
Gender	Men	141	47		
	Women	159	53		
Age (years)	18-29	18	6	21.096 ***	<.001
	30-44	60	20		
	45-59	143	48		
	≥ 60	79	26		
Education	None	60	20	54.836***	<.001
	Primary	134	48		
	Secondary	93	31		
	University	13	4		
Experience in maize farming (years)	≤ 5	23	8	3.212	0.201
	5-10	40	13		
	≥ 11	237	79		
Household size (People)	≤ 5	108	36	0.552	0.907
	6-9	145	48		
	10-14	38	13		
	≥ 15	9	3		
					

Table 2 presents the socioeconomic characteristics of the respondents.

**Table 2:** Socioeconomic characteristics of maize farmers in the Katangese Copperbelt.

Characteristics	Category	Frequency	Percentage	Gender differences	
				χ2	*p*-value
Member of farmers' groups	Yes	52	17,3	0.556	0.456
	No	243	82.7		
Members of VSLAs+	Yes	139	46.3	0.384	0.456
	No	161	53.7		
Farmers received financial support	Yes	59	19.7	5.06	0.024*
	No	241	80.3		
Land tenure	Owner	170	56.7	1.812	0.404
	Rental	89	29.7		
	Temporary use	41	13.7		
Cropland size (hectares)	< 0.5ha	82	27.4	26.259	<.001***
	0.5ha	97	32.3		
	1 ha	75	25		
	≥ 1ha	46	15.3		
Off-farm job	Yes	90	30	3.778	0.052*
	No	210	70		
Farmer grows other crops	Yes	264	88	0.01	0.977
	No	36	12		
Farmer recently attended training on agriculture (2020-2023)	Yes	78	26	0.776	0.378
	No	222	74		

+ : VSLAs: Village Savings and Loan associations.

### 3.2 Types of seeds and sources of information on improved maize varieties

**Fig. 1:**
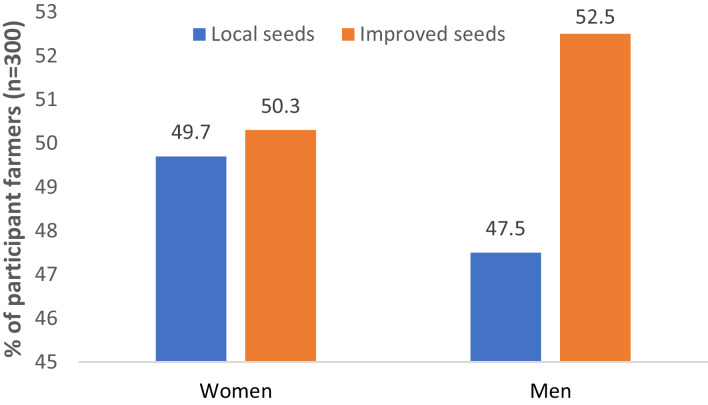
Types of seeds used by men and women farmers in the study area.

Fig. 1 shows the types of seeds used by smallholder farmers in the study area.

Table 3 presents the sources of information used by farmers in the Katangese Copperbelt regarding improved maize seeds.

**Table 3:** Sources of information used by farmers on improved maize seeds.

Sources of information on improved maize seeds	Frequency	Percent
Market (input retailers or seed suppliers)	88	29.3
Fellow farmers	187	62.3
Research Institutions	5	1.7
Farmers’ organizations	16	5.3
Government extension officer	4	1.3

### 3.3 Supply Channels used by smallholder farmers to access Improved Maize Seeds

**Fig. 2:**
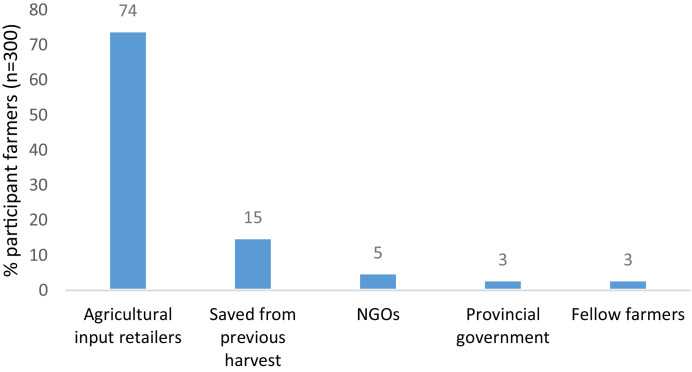
Supply Channels used by smallholder farmers to access improved maize seeds.

The supply channels smallholder farmers use to access or buy improved maize seeds are shown in Fig. 2.

### 3.4 Fear of climate change and adoption of maize-improved seeds among farmers

**Fig. 3:**
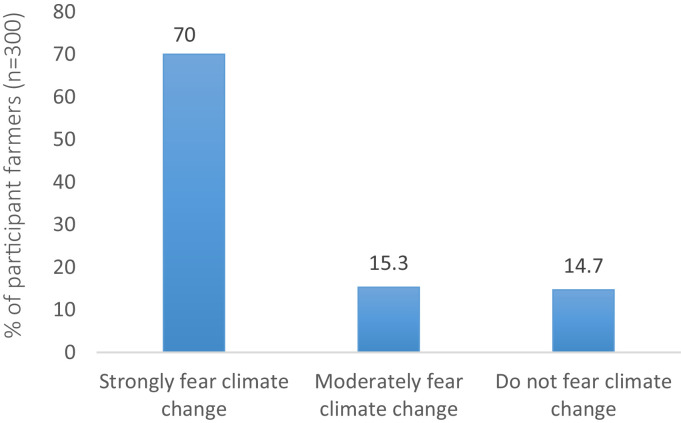
Farmers' level of concern about the impacts of climate change on maize production.

Fig. 3 shows the level of concern among farmers regarding the threats that climate change poses to maize production in the Katangese Copperbelt

**Fig. 4:**
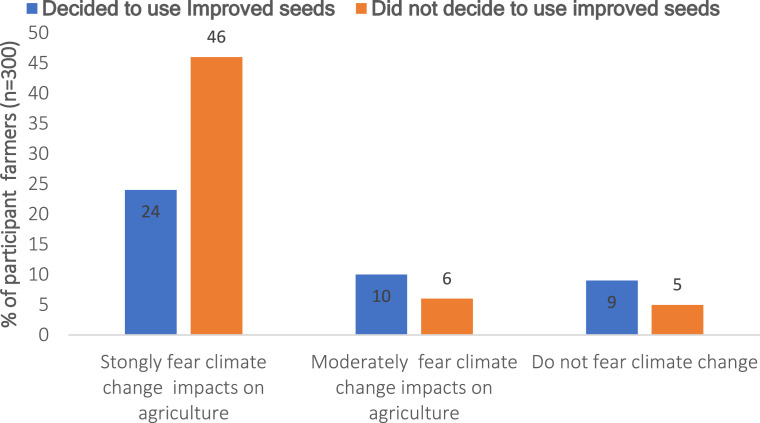
Fear of climate change and adoption of maize-improved seeds among farmers.

Fig. 4 shows the proportion of smallholder farmers in the Katangese Copperbelt who chose to use improved maize seeds over the past three years due to concerns about climate change, highlighting a significant difference among respondents (χ² = 23.358, *p* < 0.001).

### 3.5 Drivers of the adoption of maize-improved seeds in the Katangese Copperbelt

Table 4 shows the socioeconomic and institutional factors influencing the adoption of maize-improved seeds among smallholder farmers in the Katangese Copperbelt.

**Table 4:** Determinants of the adoption of maize-improved seeds in the Katanga Copperbelt.

Variables (predictors)	Coefficient	Standard Errors	Wald	Significance	Exp(B)
Gender	0.292	0.34	0.74	0.39	1.339
Farmer’ age	0.089	0.22	0.162	0.687	1.093
Education level	−0.836	0.251	11.061	<0.001***	0.433
Membership in farmers’ organization	1.063	0.441	5.817	0.016*	2.896
Membership in VSLAs+	−0.136	0.316	0.185	0.667	0.873
Financial support	1.16	0.424	7.485	0.006**	3.19
Off-farm employment	1.514	0.38	15.899	<0.001***	4.545
Experience in farming	0.072	0.312	0.053	0.818	1.074
Cropland size	0.064	0.164	0.153	0.695	1.066
Land ownership	−0.326	0.224	2.115	0.146	0.722
Other crops	−1.744	0.633	7.589	0.006**	0.175
Information on improved Seeds	−0.422	0.142	8.875	0.003**	0.656
Fear of climate change	−0.612	0.243	6.328	0.012*	0.542
Constant	−1.212	1.882	0.415	0.52	0.298
					

+ : VSLAs: Village Savings and Loan Associations., Nagelkerke R Square=50.2 %, overall percentage correctly predicted:80.3 %, *** significant at 1 %. ** Significant at 5 %,* Significant at 10 %.

### 3.6 Reasons and motivations for adopting improved maize seeds among farmers

**Fig. 5:**
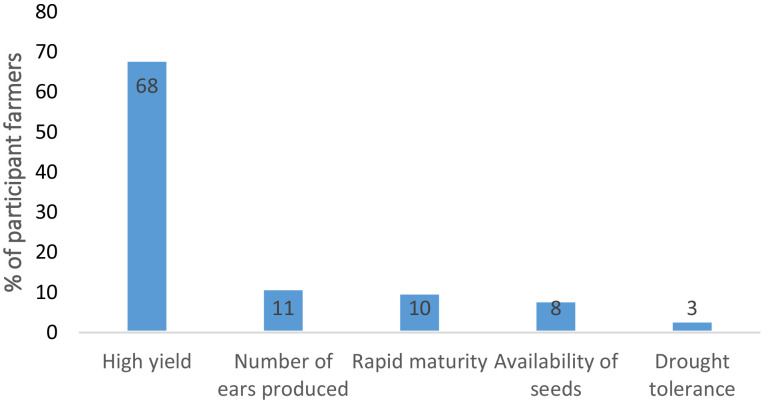
Reasons and motivations for adopting improved maize seeds among farmers.

Fig. 5. reveals the reasons behind the adoption of maize-improved seeds among smallholder farmers in the Katangese Copperbelt.

### 3.7 Barriers to the adoption of maize-improved seeds among smallholder farmers

**Fig. 6:**
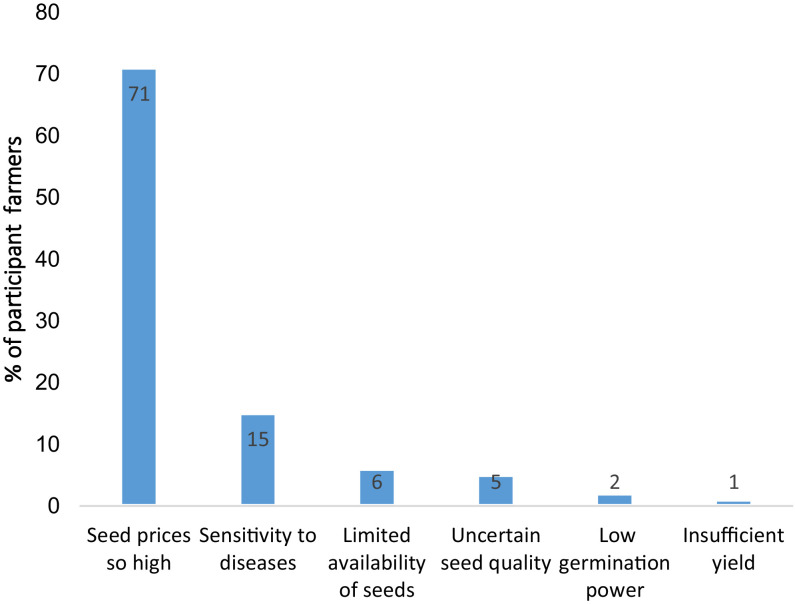
Barriers to the adoption of improved maize seeds among smallholder farmers in the Katanga Copperbelt.

The factors that prevent farmers from adopting maize-improved seeds are shown in Fig. 6.

### 3.8 Measuring farmers' confidence in recommending improved maize seeds to their Peers

**Fig. 7:**
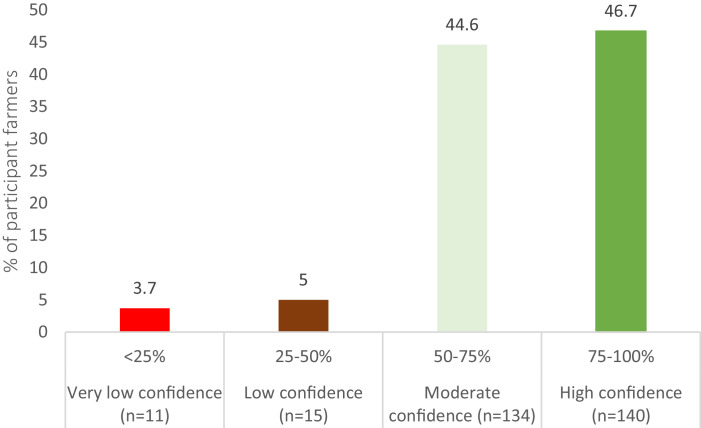
Distribution of farmers based on their confidence to recommend improved maize seeds to peers.

In the Democratic Republic of Congo, most farmers rely on peers for agricultural information due to the limited effectiveness of public extension services [7,8]. Fig. 7 shows the distribution of farmers based on their confidence in recommending improved maize seeds to their peers.

## 4 Experimental Design, Materials, and Methods

A survey questionnaire was used to collect data through face-to-face interviews with 300 smallholder farmers growing maize, using Abate et al.’s (2022 ) sample size determination formula [[5], [6], [7], [8]]. The respondents were randomly selected from Kafubu, the locality of Sambwa, and villages located along the section of National Road N^o^. 1 leading to Kasumbalesa (all in the territory of Kipushi). The survey was conducted between April and July 2024 with the help of five enumerators (third-year Agronomy students from the University of Lubumbashi). The questionnaire was designed in French (the official language of the Democratic Republic of the Congo), and interviews were mostly conducted in Kiswahili, a local language spoken and understood by most of the farmers. The submitted survey questionnaire was translated into English per the journal's requirement. The collected data were entered into a Microsoft Excel sheet (Raw data on improved maize seeds adoption in Katanga .xlsx). The dataset and the codebook used are available on Mendeley Data: https://data.mendeley.com/datasets/by7b2dj2fh/1).

The data were cleaned by removing missing entries and analyzed using the SPSS Statistical Package, Version 22. Descriptive analysis and a Chi-square test were employed to compare male and female respondents' demographic and socioeconomic profiles. To identify the predictors of improved maize seed adoption, we analyzed demographic and socioeconomic variables using a binary logistic regression model. A significance level of α < 0.05 was used as the criterion for statistical significance.

## Limitations

The first limitation was related to the availability of agricultural statistics, which hindered the acquisition of accurate data on the exact number of maize producers in Haut-Katanga. With this in mind, we initially planned to survey 385 farmers—an acceptable and recommended sample size for an unknown population, considering a 95 % confidence interval and a 5 % margin of error [[6], [7], [8], [9]]. However, due to incomplete questionnaires, the reluctance of many participants to answer, and missed appointments caused by the farmers' busy schedules, our sample size was ultimately reduced to 300 respondents.

Although numerous varieties of maize have been distributed in the Katangese Copperbelt region [2,3], many farmers do not recall the names of the varieties they cultivate and struggle to differentiate improved from local seed varieties. Another limitation is that the dataset does not include the qualitative data collected from focus groups. These qualitative insights reveal farmers' expectations of seed suppliers, plant breeders, and local government. They also shed light on how pest outbreaks, drought, poor distribution, and interruptions in rainfall during the maize-growing season discourage farmers from investing in large-scale maize production.

Lastly, in the study area where security and trust among the population have deteriorated, farmers were reluctant and cautious with interviewers regarding questions about their income and other assets. This hindered the determination of the proportion of their income allocated to maize production, especially the cost of improved seeds.

## Ethics Statement

We obtained written research authorization N^o^.Ref/FAC/AGRO/308/2024 from the University of Lubumbashi to survey farmers. Surveyed Farmers provided oral consent confirming their willingness to participate in the survey. The study does not involve human or animal parts or related experiments.

## Credit Author Statement

Mushagalusa A. Balasha: Conceptualization, Methodology, Data curation, Visualization, Writing – original draft. Angelo Flore: Conceptualization, data collection, Data curation, review; Mazinga Michel: Conceptualization, Research supervision, review & editing; Nyumbaiza Alex: Conceptualization, Methodology, Research supervision, Writing – review & editing.

## Declaration of Generative AI Use

The authors used Grammarly to enhance the language and ensure grammatical accuracy throughout the manuscript.

## Data Availability

Mendeley DataDataset on Improved Maize Seeds Adoption in the Katangese Copperbelt (Original data)
